# Can Exercise Capacity Assessed by the 6 Minute Walk Test Predict the Development of Major Adverse Cardiac Events in Patients with STEMI after Fibrinolysis?

**DOI:** 10.1371/journal.pone.0099035

**Published:** 2014-06-06

**Authors:** Ayman K. M. Hassan, Salwa R. Dimitry, George W. Agban

**Affiliations:** Department of Cardiovascular Medicine, Faculty of Medicine, Assiut University, Assiut, Egypt; Emory University, United States of America

## Abstract

**Background:**

To assess the added value of the 6 minute walk test distance (6MWTD) in the risk-stratification methods for patients with ST -segment elevation myocardial infarction (STEMI) treated with fibrinolysis.

**Methodology/Principal Findings:**

This is a prospective cohort study of one hundred consecutive patients with STEMI, who had received fibrinolysis, at Assuit University Hospital. All patients underwent 6MWT pre- discharge and were followed up for 3 months to monitor the incidence of major adverse cardiac events (MACE). Patients were divided into 3 groups according to the level of 6MWTD (level I>450 m, level II = 300–450 m and level III<300 m). Among the study population, the median 6MWT distance was 370 meters (interquartile range 162–462). The mean age was 60.9±10.7 years, 71.9% of them were males, 2/3 had anterior MI. only 10.5% had successful thrombolysis. Compared to patients in level I (>450 m), patients in level III (<300 m) were more likely to have clinical risk factors as hypertension, diabetes and impaired renal function. The patient's mean TIMI score was 3.4±2.2, the mean GRACE score was 150.5±27.7. There was a significant negative correlation between the 6 MWTD and GRACE risk score (r = −0.80, p<0.001). At 3 months of follow-up, 51% had MACE including 16% were dead. Multivariate logistic regression analysis identified that the GRACE risk score and 6MWT distance levels were the best predictors of the MACE at 3 month of follow up. The incidence of MACE was 4 times higher in patients with high GRACE risk score who couldn't walk more than 300 meters (OR = 4.66, 95% CI = 1.1–14.5, p = 0.006).

**Conclusions/Significance:**

In patients with STEMI treated with fibrinolysis, the addition of 6MWTD assessment pre-discharge to the traditional GRACE risk score improved the risk prediction of cardiovascular events at 3 month follow up.

## Introduction

The six-minute walk test (6MWT) is a simple, easy-to-perform, low tech, safe and well established self-paced assessment tool to quantify functional exercise capacity in different patients categories [1]. The 6MWT is performed by instructing the participant to march as fast as possible (without running) on a horizontal surface in 6 minutes, the distance walked (6MWD) is recorded [1].

The 6MWD is a good predictor for morbidity and mortality in patients with heart failure [1]–, pulmonary hypertension [7], and pulmonary disease [8]. Normal values available for the 6MWD are based on different adult cohorts [9], [10]. However, there is no evidence regarding the ability of the 6MWT to predict outcomes in patients with ST-elevation myocardial infarction (STEMI).

Despite the improvement of different therapeutic modalities for patients with STEMI, major adverse cardiac events (MACE) are still 8.7% [11]. The prognostic models based on traditional cardiovascular disease risk factors do not fully explain the risk of future cardiovascular events in these patients. This clarifies why various risk scores have been introduced for STEMI patients with time. In the era of fibrinolysis, several different clinical scores have been used, including the GRACE [12], PAMI [13] and TIMI-STEMI [14] scores. For the current era where primary percutanious coronary intervention (PPCI) is the gold standard, new risk scores have been introduced including Zwolle score (Zs) [15], CADILLAC score[16], EuroSCORE and SYNTAX scores [17]. However, not all patients had the facility to do PPCI. So for those undergoing fibrinolysis and cannot proceed for PPCI due to financial restraints, we still need to risk stratify these patients pre-discharge. In Upper Egypt, we need to provide our patients with the best available and affordable treatment modality.

Exercise treadmill testing although provides information regarding prognosis in STEMI patients, but it is woefully underutilized [18]. This can be explained partly by patients overly cautious or reluctant to participate in exercise after STEMI, and also testing is expensive, not widely available, and time consuming.

In the present study, we evaluated the ability of the 6MWT to predict MACE in patients with STEMI treated with fibrinolysis.

## Methods

### Ethics Statement

The study protocol was approved by the ethical committee of Assuit faculty of medicine. A written informed consent was obtained from all participants. The consent form was designed with an explanation on the purpose and conduction of this research project. This form was to be explained to each participant; then a written consent was given. Participation was only proceeded after written consent of the participant. The full text of the form was approved by the Ethical Review Committee of Assuit faculty of medicine.

### Patient's selection

Patients were included if they met the following criteria: (i) they presented typical anginal pain lasting for >30 min; (ii) there was ST-segment elevation of at least 1 mm in at least two contiguous electrocardiography (ECG) leads or new onset of complete left bundle-branch block; and (iii) they had Fibrinolysis. Exclusion criteria were absolute contraindication for fibrinolysis, primary PCI, morbid obesity or handicapped patients who cannot walk, chronic obstructive pulmonary disease, patients with clinical hemodynamic or electrical instability.

### Study design

This is a prospective cohort study. Between September 2011 and March 2012, 369 patients were enrolled in the coronary care unit of Assuit university hospital (AUH). We were not able to offer the 6MWT to all patients for logistical reasons (e.g., not enough time prepare the protocol, busy nurses, obstruction of the 6MWT corridor, study staff unavailable). Of the 115 patients who were offered the 6MWT, 4 were unable to complete the 6MWT (recently experiencing unusual chest pain, or did not think they were able to walk for 6 minutes due to shortness of breath or musculoskeletal barrier), 10 refused, and 1 had incomplete data. These 15 were excluded from this analysis because they did not complete the 6MWT, leaving 100 participants for this analysis.

### Study population

We enrolled 100 consecutive patients, who fulfilled the inclusion and exclusion criteria for this study. Demographic characteristics, medical history, and smoking status were assessed. We measured weight, resting blood pressure and heart rate. The mean time in minutes from symptoms onset (the maximum intensity of the pain felt by the patients) to the start of fibrinolysis in CCU (symptoms –to-needle time) was documented. Before taking the fibrinolysis, we calculated the TIMI risk score [14], which include (age (years), weight (kg), DM, HTN, UA, heart rate (bpm), systolic BP (mmHg), ST elevation, LBBB, Killip class and time to treatment) and the GRACE risk score which include (age (years), heart rate (bpm), systolic BP (mmHg), creatinine (mg/dL), Killip class, cardiac arrest at admission, elevated cardiac markers and ST segment deviation) [12], [20], [21]. After initial event, all patients received asprin (150 mg/day) indefinitely, clopidogrel (75 mg/day) for one year, other medications including beta blocker, angiotensin converting enzyme inhibitors, nitrates and statins, were prescribed according to STEMI standard guidelines [22].

### Six-Minute Walk Test (6MWT)

The 6MWT was administered according to standard guidelines just before the discharge of the patients from CCU [1]. A single walk test without practice was administered. Participants were instructed to walk continuously on a CCU corridor 40 m. in length, covering as much ground as they could during six minutes. The test was performed under the control of a physician who encouraged the patients using phrases like ‘You are doing well’ or ‘You are doing a good job’. At the end of the 6-min, the physician measured the total distance walked by the patient. On the basis of the distance walked, performance was grouped into three different levels (level I>450 m, level II = 300–450 m and level III<300 m) [1], [23].

### Laboratory data

Creatine kinase (CK) activity (Dimension Xpand Plus, Siemens Diagnostics; upper limit of normal (ULN) = 308 U/L). The cumulative release of CK in the first 48 hours was calculated as a measure of infarct size in each patient by an investigator blinded to the assigned treatment [24]. Serum creatinine level (Dimension Xpand Plus, Siemens Diagnostics; upper limit of normal (ULN) = 115 µmol/L).

### Electrocardiographic data analysis

ST segment elevation was measured manually 20 ms after the end of the QRS complex (the J point) using a hand-held caliper. The sum of ST-segment elevation in leads V1 to V6, I, and aVL was added to the sum of ST-segment depression in leads II, III, and aVF for anterior MI. For inferior MI, the sum of ST-segment elevation in leads II, III, and aVF (and I, aVL, V5, and V6; if present) were added to the sum of ST-segment depression in leads V1 to V4. All ECGs were collected and analyzed by an investigator blinded to the assigned treatment. Total ST-segment deviation at inclusion was compared to that taken within 90 minutes after PPCI. ST-segment resolution ≥50% of the initial ST-segment deviation was calculated [25], [26].

### Outcome Ascertainment

All patients were followed up for 3 months. Clinical outcome was evaluated through the monitoring of major adverse cardiac events (MACE) occurring at any time during the follow up. Death was defined as “all-cause” death at follow-up. Myocardial re-infarction was defined using standard diagnostic criteria [27]. Heart failure was defined as hospitalization or emergency department visit for signs and symptoms of heart failure. The primary outcome was incidence of MACE during follow up. Secondary outcomes were the individual components of heart failure, myocardial infarction, and death from any cause.

### Statistical analysis

Patients were divided into 3 groups according to level of 6MWT distance. Baseline patient's characteristics were compared using analysis of variance (ANOVA) for continuous variables, a chi-squer (*X^2^*) test for dichotomous variables, and fisher exact test for dichotomous variables with fewer than 5 patients in a category. Correlation between different variables was evaluated using Pearson correlation coefficient analysis. Univariate analysis was performed using *X^2^*test with Yates' correction when necessary. Multivariate logistic and linear regression analyses were performed using all potentially relevant variables to identify baseline independent predictors of MACE. All p-values are two-tailed, and statistical significance was defined if p<0.05. All analyses were performed with SPSS version 16.0 statistical software (SPSS Inc., Chicago, IL, USA).

## Results

### Patient characteristics

The study population consists of 100 patients with STEMI received fibrinolysis. Patients were divided into 3 groups according to level of 6MWT distance. Baseline characteristics of our study population are summarized in table 1. The 3 groups were comparable, with no significant differences in baseline characteristics. In our study the patient's mean age was 60.9±10.7 years (ranging from 25–83 years) and the patient's mean weight was 73.7±9.7 (ranging from 49–100 kg).

**Table 1:** Baseline characteristics of the study population by levels of sex-minute walk test distance.

		6MWT levels (m)			
	All group	Level I (>450 m)	Level II (300–450 m)	Level III (<300 m)	P
*Number of pt.*	*10 pt*	40 pt	32 pt	28 pt	
***Demographics***					
Age (years)	60.9±10.7	58.5±11	62.5±10	62.3±8	NS*
Gender (Males)	72 (72%)	26	28	18	NS†
Smoking	47 (47%)	18	17	12	NS†
***Medical history***					
Hypertension	46(46%)	16	10	20	0.08†
Diabetes mellitus	35 (35%)	5	11	19	0.01†
Known to be Ischemic	33 (33%)	9	12	12	NS†
previous PCI	4 (4%)	0	2	2	NS†
previous CABG	7 (7%)	2	2	3	NS†
Dyslipidemia	25 (25%)	7	10	8	NS†
Previous admission to CCU	25 (25%)	9	7	9	NS†
Family history	16 (16%)	4	5	7	NS†
***Clinical measurements***					
Weight (kg)	73.7±9.7	73.5±8	72.4±9	75.2±12	NS*
Systolic BP (mmHg)	134±30	131±19	122±62	126±41	NS*
Diastolic BP (mmHg)	77±25	80±14	74±33	77±26	NS*
Pulse (bpm)	88±24	89±18	81±31	91±23	NS*
Symptoms -to-Needle Time (min)	276±37	169±35	318±75	332±85	NS*
Arrest on admission	3 (3%)	0	1	2	NS†
***Infarction location***					NS†
Anterior MI	67 (67%)	15	24	26	
Inferior MI	14 (14%)	5	2	7	
Infro-posterior MI	16 (16%)	7	5	4	
Lateral MI	3 (3%)	2	1	0	
**KILLIP SCORE**					NS†
SCORE 1	77 (77%)	33	25	19	
SCORE 2	18 (18%)	7	4	7	
SCORE≥3	5(5%)	0	2	3	
Duration till doing 6MWT (Days)	3.5±2	3.2±1	3.5±1	4.2±2	NS*
6MWT by meters	329±153	466±25	352±40	106±53	<001*

Data are presented as mean ± standard deviation, number (%) of patients.

*Compared using ANOVA test.

† Compared using Chi-square or Fisher exact test. BP =  blood pressure; MI =  myocardial infarction; TIMI =  Thrombolysis In Myocardial Infarction; CK =  creatine kinase; 6MWT =  sex-minute walk test; PCI =  percutaneous coronary intervention; CABG = coronary artery bypass graft; NS = not significant.

Among the study population, the median 6MWT distance was 370 meters (interquartile range 162–462). Compared to patients in level I (>450 m), patients in level III (<300 m) were more likely to have clinical risk factors of hypertension, diabetes and impaired renal function test (Table 1). Electrocardiographic and infarct size results are summarized in TABLE 2, 60% of our study population had failure of thrombolytic therapy with ST segment resolution <50% at 90 min post fibrinolysis. The baseline levels of CK were similar in all groups. The infarct size assessed by the cumulative 48-h CK release was not statistically different across groups. Patients in level III had more failure of thrombolytic therapy by ECG analysis and higher risk grade in GRACE score (Table 2).

**Table 2:** Laboratory, electrocardiographic and risk scoring results of the study population by levels of sex-minute walk test distance.

		6MWT levels (m)			
	All group	Level I (>450 m)	Level II (300–450 m)	Level III (<300 m)	P
*Number of pt.*	*100 pt*	40 pt	32 pt	28 pt	
*Basal CK (U/L)*	*1616±195*	*1454±262*	*1211±270*	*2160±437*	*NS* *
*Cumulative 48-h CK release (U/L)*	*9085 (5976–14593)*	*8174 (4135–13482)*	*8465 (4558–14466)*	*9296 (6013–15012)*	*NS* *
***Serum creatinine levels (µmol/L)***					***0.002*** *
*0.4–O.79*	*21(21%)*	*14*	*4*	*3*	
*0.8–1.1*	*46(46%)*	*22*	*15*	*9*	
*1.2–1.59*	*19(19%)*	*3*	*7*	*9*	
*1.6–1.99*	*7(7%)*	*0*	*3*	*4*	
*2–3.99*	*7(7%)*	*0*	*3*	*4*	
***Success of fibrinolysis using ECG ST resolution***					***0.04*** †
*<50%*	*60 (60%)*	*12*	*22*	*26*	
*50–75%*	*30 (30%)*	*14*	*14*	*2*	
*>75%*	*10 (10%)*	*6*	*3*	*1*	
*TIMI risk score*	*3.4±2*	*2.9±2*	*3.6±2*	*3.6±2*	*NS* *
***GRACE risk score***	*147±29*	*123±17*	*161±27*	*166±21*	***<0.001*** *
*Low risk (49–125)*	*25 (25%)*	*25*	*0*	*0*	
*Intermediate risk (126–154)*	*33 (33%)*	*15*	*14*	*4*	
*High risk (155–319)*	*42 (42%)*	*0*	*17*	*25*	

Data are presented as mean ± standard deviation, number (%) of patients.

*Compared using ANOVA test.

† Compared using Chi-square or Fisher exact test. TIMI =  Thrombolysis In Myocardial Infarction; CK =  creatine kinase; 6MWT =  sex-minute walk test; *GRACE* =  Global Registry of Acute Coronary Events; NS = not significant.

**Figure 1:**
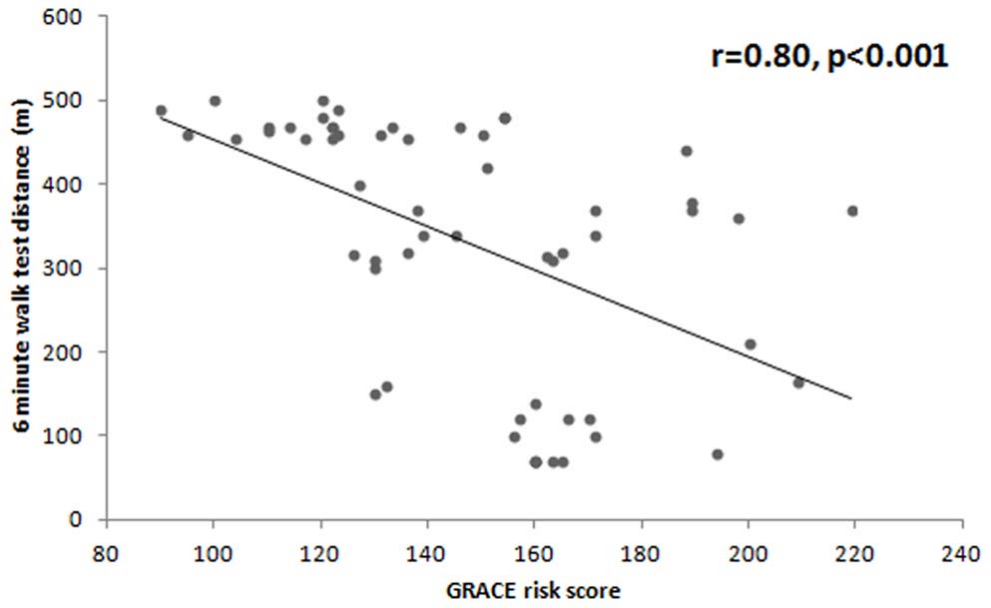
Six-minute walk test distance by GRACE risk score.

Six-minute walk test was compared with GRACE risk score. There was a significant negative correlation between 6 MWT distance and GRACE risk score (r = −0.80, p<0.001) (Figure 1).

**Figure 2:**
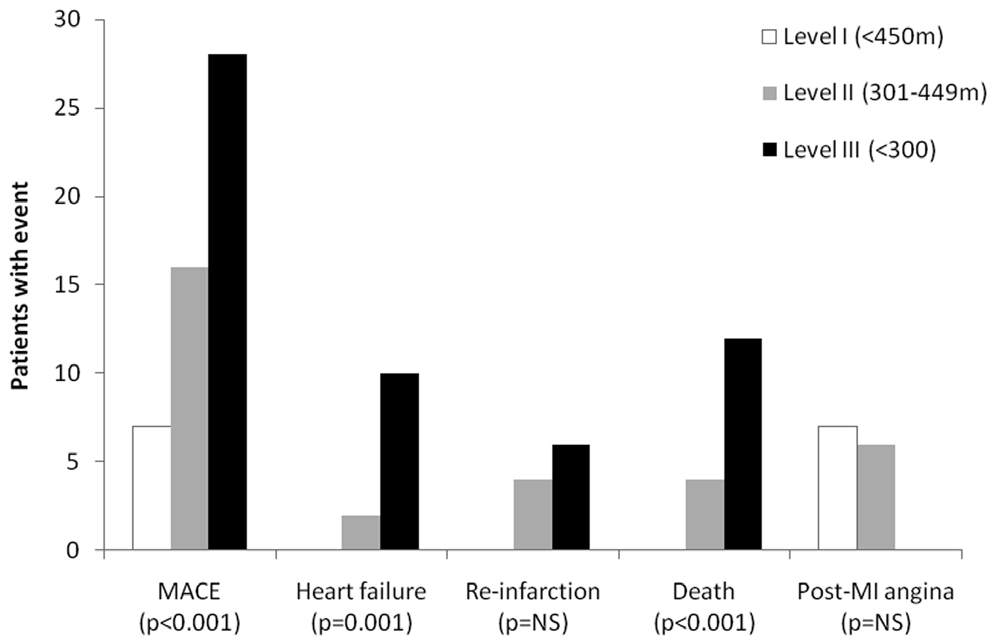
Cardiovascular events by levels of six-minute walk test distance.

All patients were followed up for 90 days. Cumulative MACE occurred in 51 patients. 12 patients had heart failure hospitalization, 10 patients had re-infarction, 13 patients had post MI angina and 16 patients died (table 3). Incidence of MACE was higher in patients with level III compared to other levels of 6MWT distance (Figure 2).

**Table 3:** Clinical outcomes and complications at 90 days follow-up in the study population by levels of sex-minute walk test distance.

		6MWT levels (m)			
	All group	Level I (>450 m)	Level II (300–450 m)	Level III (<300 m)	P
*Number of pt.*	*100 pt*	40 pt	32 pt	28 pt	
MACE	51 (51%)	7	16	28	<0.001†
Heart failure	12 (12%)	0	2	10	0.001†
Re-infarction	10 (10%)	0	4	6	0.09†
Post-MI angina	13 (13%)	7	6	0	NS†
Death	16 (16%)	0	4	12	<0.001†

Data are presented as number (%) of patients.

† Compared using Chi-square or Fisher exact test. MACE =  major adverse cardiac events; MI =  myocardial infarction.

### Prediction of MACE after STEMI using logistic regression analysis

Success of fibrinolysis, Anterior MI, Infarction size using 48 h CK and TIMI risk score were positively related to incidence of MACE (Table 4). Symptom to Needle time was not significantly related to MACE.

**Table 4:** Logistic regression analysis for prediction of MACE.

	Univariable analysis			Multivariable analysis		
	OR	95%CI	P	OR	95%CI	P
Age	**1.03**	**0.98–1.09**	**0.26**	**-**	**-**	**-**
Male gender	**0.52**	**0.16–1.61**	**0.27**	**-**	**-**	**-**
Smoking	**0.65**	**0.22–1.91**	**0.44**	**-**	**-**	**-**
Hypertension	**0.99**	**0.33–2.88**	**0.98**	**-**	**-**	**-**
Diabetes	**0.79**	**0.25–2.44**	**0.68**	**-**	**-**	**-**
Previous MI	**0.54**	**0.05–3.58**	**0.44**	**-**	**-**	**-**
Dyslipidemia	**1.16**	**0.53–2.50**	**0.70**	**-**	**-**	**-**
Positive family history	**0.95**	**0.44–2.07**	**0.91**	**-**	**-**	**-**
Killip class ≥2	**2.21**	**0.19–25.14**	**0.91**	**-**	**-**	**-**
Symptom to Needle time	**1.00**	**0.99–7.74**	**0.88**	**-**	**-**	**-**
Anterior MI	**2.04**	**0.9–4.5**	**0.07**	**-**	**-**	**-**
Infarction size using CK	**0.93**	**0.88–0.99**	**0.07**	**-**	**-**	**-**
**Success of fibrinolysis using ECG**	**0.94**	**0.88–0.99**	**0.04**	**0.96**	**0.86–1.01**	**0.11**
**GRACE risk score**	**7.23**	**3.4–15.1**	**0.004**	**2.46**	**1.15–5.90**	**0.07**
**TIMI score**	**3.08**	**1.1–8.1**	**0.07**	**1.33**	**0.86–3.90**	**0.12**
**6MWT risk score**	**7.14**	**3.3–17.1**	**0.005**	**2.66**	**1.15–4.90**	**0.07**
**Combination of GRACE+6MWT**	**8.14**	**3.3–19.1**	**<0.001**	**4.66**	**1.15–14.90**	**0.006**

MI: myocardial infarction, PPCI: primary percutanious coronary intervention, TIMI =  Thrombolysis In Myocardial Infarction; CK =  creatine kinase; 6MWT =  sex-minute walk test; *GRACE* =  Global Registry of Acute Coronary Events.

GRACE risk score and 6MWT distance levels were the strongest univariable predictors and the only significant predictors in multivariable logistic regression analysis for the incidence of MACE (Table 4). The incidence of MACE were 2 times higher in patients with a level III 6MWTand GRACE risk scores (OR = 2.66, 95% CI = 1.15–4.9 & OR = 2.46, 95% CI = 1.15–5.9). According to temporal sequence of events, GRACE risk score can be assessed early just after STEMI diagnosis, but 6MWT score is only done pre-discharge. So after combining both scores in the multivariable analysis model, we identified only combination of 2 scores as an independent predictor of MACE (OR = 4.66, 95% CI = 1.1–14.5, p = 0.006). So it is reasonable to assume that adding 6MWT score to GRACE risk score will support the prediction of MACE in patients with STEMI treated with fibrinolysis.

## Discussion

In a cohort of patients with STEMI treated with fibrinolysis, we found that shorter distance walked on 6MWT was associated with higher rates of heart failure, myocardial re-infarction, and death, independent of traditional cardiovascular disease risk factors and scoring systems. The 6MWT provided additional predictive information beyond traditional risk factors and scores. The ability of the 6MWT to predict cardiovascular events was similar to traditional GRACE risk score. These findings suggest that a simple 6MWT is a useful prognostic marker for identifying STEMI patients treated with fibrinolysis at low risk who may not need further interventions.

There has been limited evidence regarding the prognostic ability of 6MWT in patients with STEMI treated with fibrinolysis. One study evaluated patients with chronic stable coronary heart disease and found 6MWT to be predictor of cardiovascular events [28]. Another study evaluated patients with recent coronary artery bypass surgery undergoing cardiac rehabilitation and found 6MWT to be a predictor of mortality [29]. Our findings extend the evidence that the 6MWT predicts cardiovascular events to patients with STEMI treated with fibrinolysis. The results of our study also expand beyond previous studies that have investigated 6MWT in patients with heart failure [1]–[6]. Although 6MWT distance did not reliably correlate with cardiopulmonary exercise testing measures in previous studies [30], [31], most studies found that 6MWT still predicted heart failure hospitalizations and death in patients with systolic heart failure [2], [5], [6].

In addition, Beatty et.al. [28] revealed that the 6MWTD can predicts MACE in a broader population of patients with stable CHD, independent of traditional risk factors and markers of cardiac disease severity. More over they suggest that 6MWT is a potential alternative to treadmill exercise testing for assessment of prognosis in those patients.

Treadmill exercise testing will remain the preferred modality for evaluating patients with suspected ischemia. However, for STEMI patients undergoing risk stratification for further intervention, the 6MWT offers potential advantages. The 6MWT can be conducted with little equipment other than a hallway marked for distance and a stopwatch. Due to the self-paced nature of the test, side effects of chest pain, dyspnea, or musculoskeletal pain are usually mild; serious adverse events have not been described [1]. Further, the 6MWT is less expensive than treadmill exercise testing. The ability of the 6MWT, a simple office-based test of functional exercise capacity, to predict outcomes in patients with stable CHD is especially relevant because the 6MWT addresses physical activity, a modifiable risk factor for secondary prevention of CHD [28]. While we have demonstrated in the present work that the 6MWTD can predict cardiovascular events in STEMI patients, however, its use for improving prognosis merits further study.

### Study Limitations

Our study is a single center prospective study, the level of 6MWTD was predetermined based on heart failure protocols, this consensus was taken mainly to allow comparison with other studies. The use of Treadmill exercise stress test as a control group was not valid in this study however, we recommend adding it in future studies. We cannot exclude the possibility of selection bias in the main cohort of participants, since many CCU admitted patients were not enrolled in the study for logistical reasons (eg. study staff unavailable, not enough time to prepare the protocol, busy nurses). Patients were excluded from the study if they were unable to walk. Thus, the results may not extend to patients with significant angina or other limitations in walking.

## Conclusions

In patients with STEMI treated with fibrinolysis, the addition of the distance walked on 6MWT pre-discharge to traditional GRACE risk score improved risk prediction of cardiovascular events at 3 month follow up.
